# Metabolic syndrome contributes to an increased recurrence risk of non-metastatic colorectal cancer

**DOI:** 10.18632/oncotarget.4166

**Published:** 2015-06-12

**Authors:** Jie You, Wen-Yue Liu, Gui-Qi Zhu, Ou-Chen Wang, Rui-Min Ma, Gui-Qian Huang, Ke-Qing Shi, Gui-Long Guo, Martin Braddock, Ming-Hua Zheng

**Affiliations:** ^1^ Department of Oncological Surgery, The First Affiliated Hospital of Wenzhou Medical University, Wenzhou, China; ^2^ Department of Endocrinology, The First Affiliated Hospital of Wenzhou Medical University, Wenzhou, China; ^3^ Department of Infection and Liver Diseases, Liver Research Center, The First Affiliated Hospital of Wenzhou Medical University, Wenzhou, China; ^4^ School of the First Clinical Medical Sciences, Wenzhou Medical University, Wenzhou, China; ^5^ Renji School of Wenzhou Medical University, Wenzhou, China; ^6^ Institute of Hepatology, Wenzhou Medical University, Wenzhou, China; ^7^ Global Medicines Development, AstraZeneca R&D, Alderley Park, United Kingdom

**Keywords:** non-metastatic colorectal cancer, metabolic syndrome, overall survival, disease-free survival, risk factor

## Abstract

**Objectives:**

Epidemiological data suggests a close link between metabolic syndrome (MetS) and non-metastatic colorectal cancer (NMCRC). However, the relationship between MetS and the outcome of NMCRC is less well understood. We aim to evaluate the impact of MetS on the prognosis in NMCRC patients.

**Methods:**

We performed a large cohort study of 1069 NMCRC patients. The Kaplan-Meier method was used to calculate the cumulative survival rate. Cox proportional hazard regression models were used to analyze the prognosis associated with MetS adjusting for clinicopathologic variables.

**Results:**

MetS was identified in 20.7% of NMCRC patients. Patients with MetS were more likely to be older, higher levels of blood glucose, triglycerides, high density lipoprotein, and uric acid than patients without MS (*P* < 0.05 for all). During a mean period of 59.6 months follow-up, patients with MetS had a statistically significantly lower rate of disease-free survival (DFS) than the patients without MetS (*P* = 0.014), especially local recurrence (*P* = 0.040). However, there was no difference in overall survival (*P* = 0.116). Multivariate analysis showed that the presence of MetS was an independent risk factor for DFS (HR = 0.733, 95%CI 0.545–0.987, *P* = 0.041), but not for OS (*P* = 0.118).

**Conclusions:**

MetS is associated with an increased recurrence risk of NMCRC.

## INTRODUCTION

Colorectal cancer (CRC) is the third most common malignant neoplasm in the world and approximately 1–2 million new cases are diagnosed each year [1]. The patients with non-metastatic CRC (NMCRC) have 5-year survival rates from 69.2% – 90.1% in the USA [2]. These patients are at ongoing risk for recurrences and long-term sequelae related to their underlying conditions, such as cardiovascular disease, diabetes mellitus (DM), all of which influence survival rates. Accordingly, intensive post-CRC follow-up and management can improve overall outcomes in NMCRC patients [3]. Therefore, it is necessary to identify risk factors which may be associated with adverse outcomes in patients with NMCRC.

Metabolic syndrome (MetS) was first introduced in 1988 [4]. It was characterized by a group of metabolic disturbances, especially insulin resistance and resultant hyperinsulinemia which can lead to an increased risk of cardiovascular diseases and type 2 DM [4]. The prevalence of MetS has been increasing worldwide, and has become a major public health problem, affecting up to 40% of adults in USA, and 9.8%–17.8% of the adults in China [5–7]. The most common components for MetS include hypertension, DM, obesity, hypertriglyceridemia and low high density lipoprotein cholesterol (HDL-C) according to the criteria from National Cholesterol Education Program Adult Treatment Panel III (ATP III) [8]. Recently, epidemiologic and clinical studies have indicated that the components of MetS are associated with CRC etiology, especially obesity and DM [9, 10]. Several studies also showed that MetS is linked with not only increased risk of developing cancer, but also an increased risk of cancer mortality [11, 12]. However, the results from studies on MetS and CRC outcomes were inconsistent [13, 14]. The objective of this study was to evaluate the effect of MetS on the prognosis of patients with NMCRC.

## RESULTS

### Baseline characteristics

A total of 1069 cases were enrolled in this study. The baseline characteristics of the NMCRC patients with and without MetS were listed in Table 1. Of these, a total of 221 patients met criteria for MetS. Upon baseline comparison, there were no statistically significant differences between the two groups with respect to gender, total cholesterol, LDL, AST, ALT, creatinine, past medical history of and smoking habit. However, the MetS group had a significantly higher incidence of hypertension, DM and obesity, higher values of BMI, glucose, TG and uric acid and lower values of HDL when compared with the non-MetS group (*P* < 0.05 for all, Table 1).

**Table 1:** Baseline characteristics of non-metastatic colorectal cancer patients stratified by MetS (*n* = 1069)

Variables	MetS Group (*n* = 221)	Non- MetS Group (*n* = 848)	*P*
**Demographic data**			
Male gender, *n* (%)	125(56.6%)	505(59.6)	0.421
Age (years)	68.8 ± 10.8	65.2 ± 13.0	<0.001
BMI (kg/m^2^)	24.5 ± 3.7	21.2 ± 2.9	<0.001
Diabetes, *n* (%)	55(24.9%)	46(5.4%)	<0.001
Hypertension, *n* (%)	117(52.9%)	182(21.5%)	<0.001
Obesity, *n* (%)	120(54.3%)	112(13.2%)	<0.001
Smoking, *n* (%)	49(22.3%)	222(26.2%)	0.232
**Preoperative laboratory data**			
Fasting glucose (mmol/dl)	7.2 ± 3.1	5.8 ± 1.8	<0.001
Total cholesterol (mmol/dl)	4.5 ± 1.2	4.4 ± 1.0	0.265
Triglycerides (mmol/dl)	2.1 ± 1.3	1.3 ± 0.8	<0.001
HDL (mmol/dl)	1.0 ± 0.3	1.2 ± 0.3	<0.001
LDL (mmol/dl)	2.6 ± 0.9	2.6 ± 0.8	0.908
AST (IU/L)	22.9 ± 22.1	20.1 ± 24.6	0.125
ALT (IU/L)	24.3 ± 16.2	24.5 ± 20.4	0.897
Creatinine (μmol/L)	72.5 ± 49.5	66.5 ± 27.1	0.088
Uric acid (mmol/L)	323.0 ± 101.4	291.4 ± 92.6	<0.001
CEA (ng/ml)	21.8 ± 103.3	19.1 ± 117.1	0.765
**Pathological data**			
Location			0.046
Right side, *n* (%)	25(11.3%)	132(15.6%)	
Sigmoid, *n* (%)	46(20.8%)	132(15.6%)	
Rectal, *n* (%)	126(57.0%)	453(53.4%)	
TNM Staging			0.471
Stage I, *n* (%)	44(19.9%)	149(17.6%)	
Stage II, *n* (%)	87(39.4%)	371(43.8%)	
Stage III, *n* (%)	90(40.7%)	328(38.7%)	
Differentiation			0.344
Well, *n* (%)	6(2.7%)	28(3.3%)	
Moderately, *n* (%)	154(69.7%)	625(73.7%)	
Poorly or undifferentiation (%)	61(27.6%)	195(23.0%)	
Well and Moderately, *n* (%)	160(72.4%)	653(77.0%)	0.153
Poorly and, *n* (%)	61(27.6%)	195(23.0%)	
Vascular invasion, *n* (%)	25(11.3%)	110(13.0%)	0.508
**Treatment**			0.005
Local treatment, *n* (%)	9(4.1%)	65 (7.7%)	
Op alone, *n* (%)	45(20.4%)	238 (28.1%)	
OP+ CTx and /or RTx, *n* (%)	167 (75.6%)	545 (64.3%)	
**Disease Recurrence**			
Local recurrence	21(9.5%)	52(6.1%)	0.077
Distant metastases	50(22.6%)	156(18.4%)	0.156

MetS = metabolic syndrome, BMI = body mass index, ALT = alanine aminotransferase, AST = aspartate aminotransferase, HDL = high density lipoprotein, LDL = low density lipoprotein, CEA = carcinoembryonic antigen, Op = operation, CTx = chemotherapy, RTx = radiotherapy.

### Tumor characteristics and treatment

Table 1 shows the tumor characteristics of the two groups according to the presence of MetS. There were no significant differences between TNM staging, tumor differentiation and CEA. The modality of treatment was different between the two groups, notably more NMCRC patients with MetS underwent chemotherapy and/or radiotherapy than patients without MetS (*P* < 0.001).

### Overall and disease-free survival analysis

#### Overall survival

**Figure 1:**
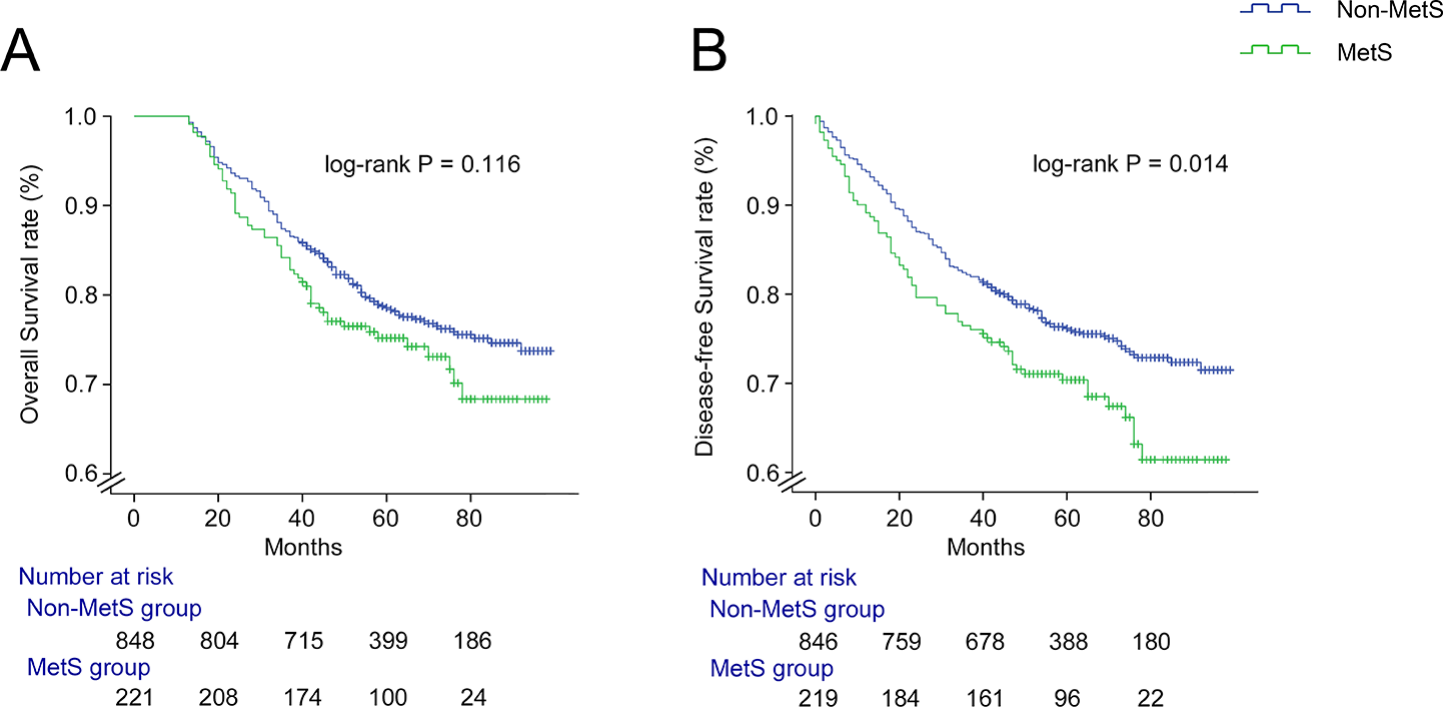
Kaplan-Meier survival curves showing overall survival **A.** and disease-free survival **B.** in non-metastatic colorectal cancer patients with and without MetS

The mean follow-up time of the cohort was 59.6 ± 21.5 months. The mean follow-up time was 56.6 ± 20.7 months in the MetS group and 60.3 ± 21.7 months in the non-MetS group (*P* = 0.023). During the follow-up period, observed patient survival was 73.8% (163/221) in the MetS group and 78.2% (663/848) in the non-MetS group. As shown in Figure 1A, there was a trend of better OS for patients with MetS compared with those without MetS, but the difference between the two survival curves was not statistically significant (*P* = 0.116). The cumulative 3-, and 5- year OS rates in the non-MetS group were 87.1%, and 80.0%, respectively, all of which were higher than OS rates of 84.2%, and 76.0%, respectively, in the MetS group (*P* = 0.230, *P* = 0.175, respectively).

#### Disease-free survival

During the mean follow-up period of 56.5 ± 25.1 months, freedom from recurrence was observed in 150 of 221 (67.9%) patients in the MetS group and 640 of 848 (75.5%) in the non-MetS group. As shown in Figure 1B, the difference between the two curves was statistically significant (*P* = 0.014). The cumulative 3-, and 5- year DFS rates in the non-MetS group were 82.2% and 77.1%, respectively, all of which were statistically higher than the DFS rates of 76.5%, and 71.0%, respectively, observed in the MetS group (*P* = 0.039, *P* = 0.044, respectively).

**Figure 2:**
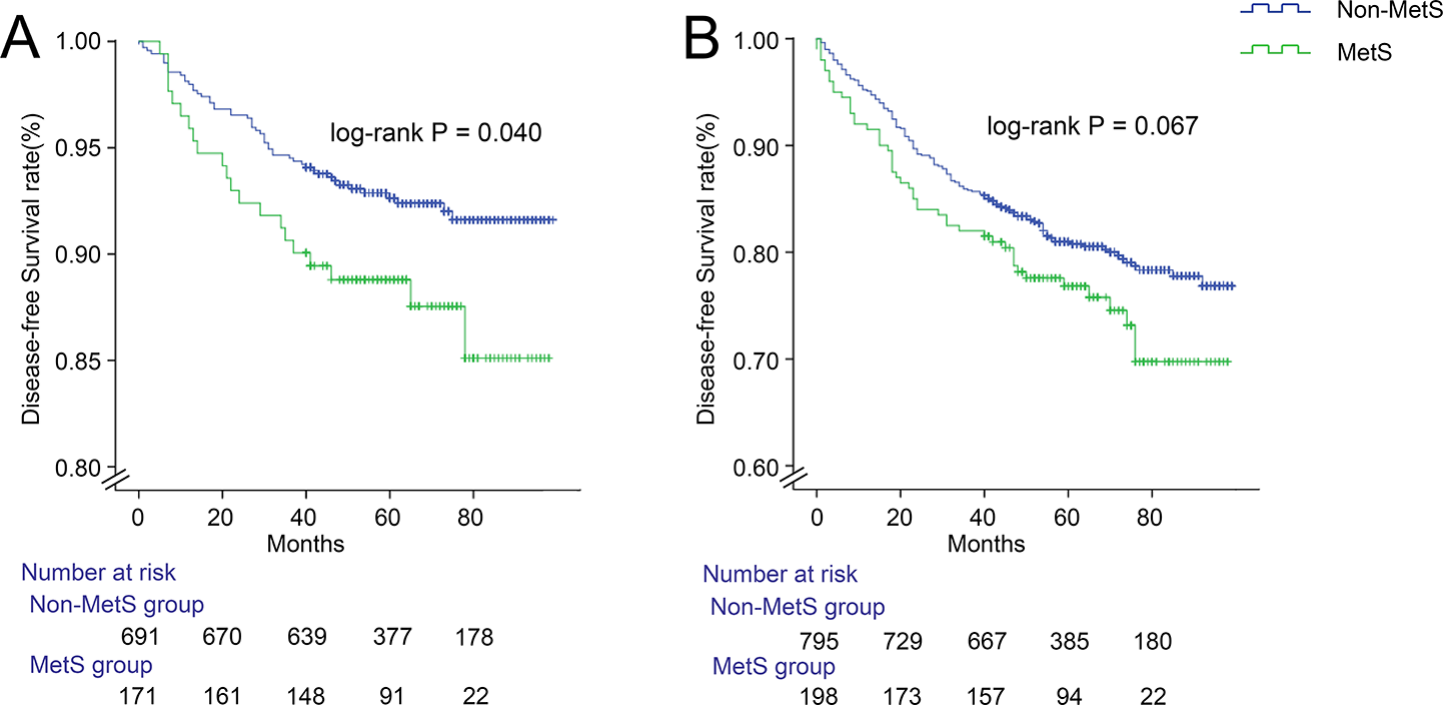
Disease-free survival stratified by local recurrence **A.** and distant metastases **B.** in non-metastatic colorectal cancer patients with and without MetS

The rates of local recurrence were 9.5% (21/221) in the MetS group and 6.1%(52/848) in the non-MetS group, the rates of distant metastases were 22.6%(50/221) in the Mets group and 18.4%(156/848) in the non-MetS group, respectively (Table 1). As shown in Figure 2A, the two curves show that the rate of local recurrence in the MetS group was significantly higher than that of the non-MetS group (*P* = 0.040). However, as shown in in Figure 2B, there was no significant difference at the rate of distant metastases between the two groups (*P* = 0.067).

#### Cox analyses of survival associated with MetS

Univariable and multivariate Cox proportional hazard models were used to identify variables associated with OS and DFS in the study population and are presented in Table 2. Fasting glucose, total cholesterol, creatinine, history of DM, hypertension and obesity were not significant predictive factors for the prognosis of NMCRC patients as determined by univariate analysis. In the multivariate Cox analysis of OS, gender, TNM staging, uric acid were independent predictive risk factors for the prognosis of NMCRC patients after adjustment for tumor differentiation, vascular invasion, CEA, total cholesterol, HDL, LDL, AST and ALT (*P* < 0.05 for all, Table 2).

**Table 2:** Cox proportional hazards regression models of risk factors associated with overall and disease-free survival among non-metastatic colorectal cancer patients (*n* = 1069)

	Overall Survival						Disease-free Survival					
	Univariable			Multivariable			Univariable			Multivariable		
	HR	95%CI	*P*	HR	95%CI	*P*	HR	95%CI	*P*	HR	95%CI	*P*
MetS	0.790	0.588–1.062	0.118				0.714	0.545–0.935	0.014	0.733	0.545–0.987	0.041
Gender	1.480	1.133–1.932	0.004	2.428	1.589–3.711	<0.001	1.428	1.115–1.829	0.005	0.695	0.533–0.906	0.007
Age	1.004	0.994–1.014	0.450				1.004	0.994–1.013	0.448			
BMI (kg/m^2^)	0.971	0.933–1.009	0.135				0.983	0.948–1.019	0.346			
<18.5	1.000						1.000					
18.5–24.9	0.662	0.483–0.907	0.010				0.785	0.581–1.060	0.114			
25–30	0.794	0.539–1.170	0.244				0.872	0.601–1.266	0.473			
>30	1.037	0.513–2.099	0.919				1.232	0.630–2.411	0.542			
Obesity	0.887	0.660–1.191	0.425				1.103	0.834–1.458	0.491			
Fasting glucose	1.009	0.956–1.064	0.744				1.004	0.955–1.057	0.863			
TNM Staging	0.315	0.243–0.409	<0.001	0.274	0.184–0.408	<0.001	0.353	0.278–0.449	0.000	0.373	0.286–0.486	<0.001
Stage I	1.000						1.000					
Stage II	1.016	0.643–1.604	0.946				0.989	0.659–1.487	0.959			
Stage III	3.208	2.117–4.862	0.000				2.812	1.936–4.085	0.000			
Differentiation	0.006	0.721–0.948	0.006				0.817	0.719–0.928	0.002			
Vascular invasion	0.625	0.452–0.864	0.004				0.624	0.460–0.847	0.003			
CEA	1.001	1.000–1.002	0.002				1.001	1.001–1.002	0.000	1.001	1.000–1.002	0.039
Total cholesterol	0.871	0.763–0.994	0.041				0.920	0.815–1.039	0.180			
Triglycerides	0.971	0.847–1.113	0.669				1.024	0.913–1.148	0.690			
HDL	0.584	0.378–0.903	0.016				0.478	0.315–0.724	0.000	0.504	0.321–0.792	0.003
LDL	0.781	0.637–0.956	0.017				0.878	0.731–1.055	0.164			
AST	1.004	1.000–1.008	0.049				1.003	0.999–1.008	(Continued )0.101			
ALT	1.006	1.001–1.010	0.010				1.005	1.000–1.009	0.049			
Creatinine	1.002	0.999–1.005	0.281				1.001	0.998–1.004	0.345			
Uric acid	0.999	0.997–1.000	0.046	0.996	0.994–0.998	0.001	0.999	0.998–1.000	0.117			
Diabetes	0.965	0.628–1.483	0.872				0.940	0.632–1.398	0.759			
Hypertension	0.903	0.685–1.191	0.469				0.819	0.635–1.055	0.122			

MetS = metabolic syndrome, CRC = colorectal cancer, BMI = body mass index, ALT = alanine aminotransferase, AST = aspartate aminotransferase, HDL = high density lipoprotein, LDL = low density lipoprotein, CEA = carcinoembryonic antigen, HR = hazard ratio, CI = confidence interval.

The presence of MetS, gender, TNM staging, differentiation, vascular invasion, CEA and HDL were significant predictive factors for DFS of CRC patients as determined by univariate analysis. In the multivariate Cox analysis of DFS, MetS, gender, TNM staging, CEA, HDL were independent predictive risk factors for the prognosis of NMCRC patients after adjusting for differentiation and vascular invasion (*P* < 0.05 for all, Table 2).

#### Subgroup analyses associated with MetS

In the subgroup analyses, the treatment options had no direct impact on patient prognosis adjusting for MetS (Table 3). However, the presence of MetS had a significant impact on DFS in patients with stage III CRC (*P* = 0.001), when stratified by TNM staging (Table 3). On consideration of the impact of BMI in the different ranges, multivariate analysis showed that BMI in the abnormal ranges (BMI < 18.5 kg/m^2^ and BMI ≥ 25 kg/m^2^) had no impact on the prognosis of NMCRC patients after adjustment for MetS for all above covariates. However, the presence of MetS had a significant impact on the prognosis for patients with BMI in the normal range (18.5–24.9 kg/m^2^) (*P* < 0.05) (Table 4).

**Table 3:** Cox proportional hazard regression analysis of overall and disease-free survival stratified by TNM staging and treatment options in non-metastatic colorectal cancer patients adjusting for MetS (*n* = 1069)

	Overall Survival			Disease-free Survival		
	HR	95% CI	*P*	HR	95% CI	*P*
Tumor location						
Colon	0.529	0.346–0.809	0.003	0.479	0.324–0.707	<0.001
Ascending colon	0.527	0.239–1.165	0.114	0.501	0.246–1.020	0.057
Descending colon	0.540	0.254–1.149	0.110	0.401	0.205–0.787	0.008
Sigmoid colon	0.501	0.252–0.996	0.049	0.513	0.265–0.992	0.047
Rectal	1.069	0.706–1.619	0.752	0.964	0.662–1.404	0.849
Gender						
Male	0.816	0.563–1.181	0.281	0.755	0.528–1.045	0.088
Female	0.72	0.441–1.175	0.188	0.634	0.408–0.986	0.043
TNM Staging						
Stage I	1.671	0.575–4.855	0.345	1.382	0.570–3.351	0.474
Stage II	0.781	0.431–1.416	0.416	0.895	0.516–1.551	0.692
Stage III	1.416	0.987–2.031	0.059	1.779	1.275–2.482	0.001
Treatment options						
Local treatment	0.608	0.207–1.780	0.364	0.475	0.179–1.262	0.136
Op alone	0.780	0.320–1.900	0.585	0.722	0.332–1.573	0.412
OP + CTx and /or RTx	0.844	0.608–1.171	0.310	0.764	0.565–1.033	0.081

MetS = metabolic syndrome, CRC = colorectal cancer, Op = operation, CTx = chemotherapy, RTx = radiotherapy, HR = hazard ratio, CI = confidence interval.

**Table 4:** Cox proportional hazards regression analysis of overall and disease-free survival from any cause associated with BMI among non-metastatic colorectal cancer patients (*n* = 1069)

BMI (kg/m^2^)	Overall Survival						Disease-free Survival					
	Multivariable^*^			Multivariable^**^			Multivariable^*^			Multivariable^**^		
	HR	95%CI	*P*	HR	95%CI	P	HR	95%CI	*P*	HR	95%CI	P
<18.5	0.839	0.303–2.323	0.736	0.890	0.708–1.119	0.319	0.942	0.781–1.136	0.530	0.935	0.756–1.155	0.531
18.5–24.9	0.793	0.700–0.899	<0.001	0.808	0.703–0.930	0.003	0.821	0.732–0.920	0.001	0.836	0.737–0.949	0.006
25–30	1.194	0.925–1.540	0.173	1.197	0.879–1.630	0.254	1.117	0.875–1.426	0.373	1.170	0.869–1.575	0.301
>30	0.937	0.744–1.178	0.576	1.182	0.678–2.061	1.182	0.947	0.770–1.165	0.605	1.189	0.815–1.735	0.368

MetS = metabolic syndrome, CRC = colorectal cancer, HR = hazard ratio, CI = confidence interval.

* Adjusted for MetS.

** Adjusted for all covariates (age, gender, MetS, TNM staging, differentiation, high density lipoprotein, uric acid, carcinoembryonie antigen).

**Figure 3:**
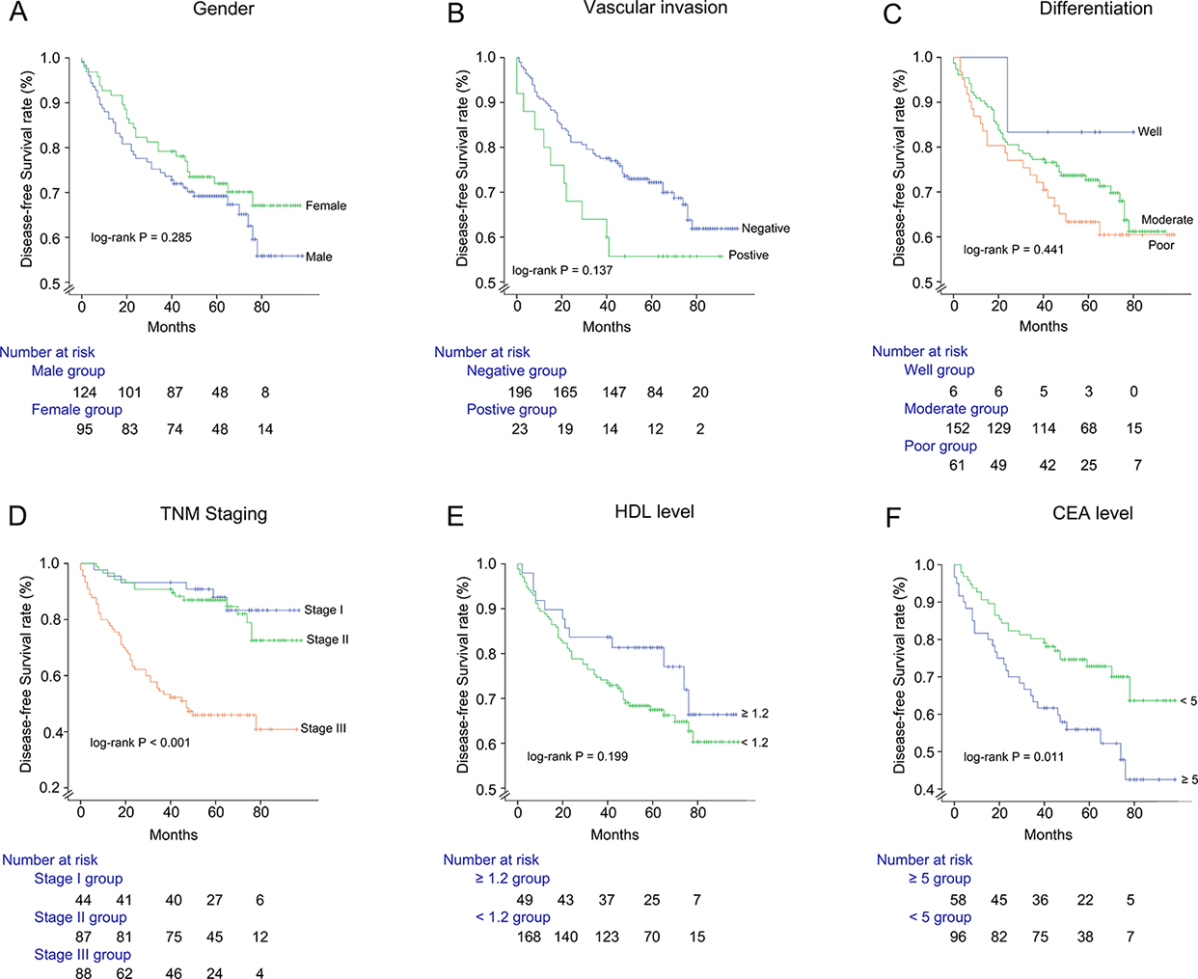
Disease-free survival of MetS group following surgical resection, stratified by gender **A.**, vascular invasion **B.**, tumor differentiation **C.**, TNM Staging **D.**, HDL level **E.**, CEA level **F.**

In the MetS subgroup, the patients were stratified according to gender, TNM stage, tumor differentiation, HDL (<1.2 vs. ≥ 1.2 mmol/dl), and CEA (<5 vs. ≥ 5 ng/ml), respectively (Figure 3). Analysis of the risk factors of DFS showed that there were significant differences in TNM staging and CEA (*P* < 0.001, *P* = 0.011, respectively) (Figure 3A–3F, respectively).

## DISCUSSION

In this cohort study, MetS was associated with the risk of recurrence (HR = 0.714, *P* = 0.014), especially local recurrence (*P* = 0.040), but not mortality. The association remained statistically significant even after adjusting for potential clinicopathogic variables (HR = 0.733, *P* = 0.041). Of individual components, only HDL was independently associated with increased risk of recurrence. Hypertension, DM and obesity were not associated with increased risk of recurrence and overall mortality in the multivariate analyses of OS and DFS. Interestingly, the subgroup analyses showed that even within the normal BMI range (18.5–24.9 kg/m^2^), there was significant association between MetS and the prognosis in NMCRC patients.

Previous epidemiological investigations have confirmed that MetS is a risk factor for CRC [15, 16], however, current studies observed an inconsistent impact of MetS and/or its components on CRC outcome [13, 14], In the report by Trevisan et al, syndrome X (defined as having high blood glucose, high blood pressure, low HDL, and high TG) was associated with a significantly increased risk of mortality from cancer in women (but not in men) [17]. Based on large-scale epidemiological data, two other reports further confirmed that the participants with MetS had significantly increased in risk of mortality from CRC compared to those without MetS, however, the independent effect was only observed in man [12, 18, 19]. Different from the above large population-scale, one study have found a positive association between MetS and risk of CRC mortality and recurrence in 507 CRC patients [14], however, another study found that MetS had no apparent effect on colon cancer outcomes [13]. Conversely, our findings are inconsistent with above conclusions regarding an increased risk of NMCRC outcomes with gender. The seemingly contradictory effect of MetS on CRC outcomes may be explained by the different definitions of MetS and the different population.

In contrast with the above inconsistent results on MetS and CRC, the studies on the each component of MetS showed a positive association with CRC incidence and mortality. A recent systematically review suggested that HDL-C was associated with decreased risk of CRC [20, 21], Other study indicated that low HDL levels tended to present more advanced lymph node stages which could predict the poor prognosis [22]. In this study, low HDL-C was associated with a risk of survival and recurrences (*P* = 0.003), which was consistent with the results for the prognostic effect of HDL-C for women with breast cancer with MetS (OR = 1.83 95%CI 1.24–2.70) [23].

It is well known that insulin is a major anabolic hormone that can stimulate cell proliferation and binding to the insulin receptor can lead to activation of the mitogen-activated protein kinase pathways [24]. The association between high levels of insulin and insulin-like growth factor-1 (IGF-1) and the risk of colon cancer has also been demonstrated. However, the mechanisms by which MetS affect the prognosis are not completely understood; high levels of insulin and IGF-1 and chronic inflammatory statuses are presumed to be important underlying factors. Considerable evidence has accumulated showing positive associations of high levels of insulin and IGF-1 and the risk of developing CRC. The previous study indicated that high levels of circulating C-peptide (a marker of insulin secretion) and lower level of IGFBP-1 were associated with increased mortality in patients with NMCRC [25]. Obesity is associated with chronic inflammatory status in CRC [26, 27], high circulating levels of C-reactive protein have been shown to be associated with poor prognosis in CRC [28–30].

MetS is a reversible condition associated with a western lifestyle [26, 31]. Physical activity, omega-3 fatty acids and some diets appear to confer some protection. Some trials have consistently showed that a Mediterranean diet can lead to regression of MetS and have a protective role regarding the development of CRC [32, 33]. Other possible adverse influence of DM or hypertension on CRC prognosis may be counterbalanced by potential protective effects of some medications, such as Metformin [34], antagonism of beta-adrenergic signaling [35] and statins [36, 37].

Our study has several limitations. First, we chose the Chinese criteria for MetS in this study. Using different criteria would likely generate slightly different incident rates of MetS that may influence the results of clinical outcomes [8, 38]. Secondly, short follow-up time (5 years) and a relatively small sample size of MetS patients (*n* = 221) may constitute other limitations of our study. However, these limitations may be partially offset by analyses of BMI, tumor location, gender, tumor staging and treatment options. A further limitation of our study may be the retrospective design which could lead to selection bias and future prospective studies may address the influence of different race or ethnic groups on the conclusions of current study.

In summary, our current study suggests that MetS, as whole, might be associated with an increased recurrence risk of NMCRC patients. The evaluation of the prognosis of NMCRC is complex, because of the different clinical combinations of the various metabolic abnormalities and the individual patient treatment strategy. Further studies are needed to assess the actual role of the individual components of MetS.

## MATERIALS AND METHODS

### Study population

In this study, we enrolled 1069 patients who underwent primary surgical resection of CRC at the First Affiliated Hospital of Wenzhou Medical University between Apr 2005 and Dec 2010. We included operable CRC patients without distant metastasis at the time of diagnosis. The exclusion criteria were as follows: i) adolescents (<18 years old), ii) patients with any history of other cancers, iii) patients surviving <1 year after operation, iv) familial adenomatous polyposis syndrome of hereditary nonpolyposis CRC (17, 21). Demographic, pre-operative laboratory and pathologic data of all patients were collected from electronic medical records and reviewed. The research protocol was approved by the Ethics Committee of the First Affiliated Hospital of Wenzhou Medical University and written informed consent was obtained from every patient.

### Diagnostic criteria of MetS

MetS was defined according to the guidelines as proposed by the Diabetes Society of Chinese Medical Association in 2004. It is defined as the presence of three or more of the following parameters: (i) body mass index (BMI) ≥ 25 kg/m^2^; (ii) anti-hypertensive drug administration and (or) systolic blood pressure ≥ 140 mmHg or diastolic blood pressure ≥ 90 mmHg; (iii) TG ≥ 1.7 mmol/L and (or) HDL < 0.9 mmol/L (male), < 1.0 mmol/L (female); and (iv) fasting plasma glucose ≥ 6.1 mmol/L or 2 h postprandial glucose ≥ 7.8 mmol/L.

### Data collection

Detailed clinical data was conducted within 2 weeks before operation. Data collection included history of smoking, alcohol consumption, history of DM and hypertension, aspartate aminotransferase (AST) and alanine aminotransferase (ALT), HDL, low density lipoprotein (LDL), fasting glucose, total cholesterol, and carcinoembryonic antigen (CEA). BMI was calculated as weight in kilograms divided by height in meters squared (kg/m^2^). Subjects were defined as obese if BMI was greater than or equal to 25 kg/m^2^.

Patients with CRC were treated primarily by surgical resection with adjuvant chemotherapy for node-positive patients and node-negative patients with adverse pathological features according to the National Comprehensive Cancer Network guidelines. Tumor staging of CRC was performed according to the sixth edition of the American Joint Committee on Cancer staging manual. Information regarding tumor location, TNM staging and histological differentiation of tumors and vascular invasion and treatment options was collected from pathological and colonoscopic sample analyses.

Patients were followed up in a post-operative outpatient schedule for every 3–6 months for 2 years, every 6 months thereafter for a total of 5 years and every 1 year thereafter. Colonoscopy and computed tomography (CT) were obtained at post-operative follow-up appointments in addition to blood analysis including CEA. Tumor recurrence such as suggested by elevated CEA, abnormal findings on colonscopy or the CT scan was defined as an earlier follow-up event. Information on death was obtained either from the patient's social security death index, outpatient medical records, or notifications from the family of the deceased. The deadline of follow-up time was June 1, 2014. Overall survival (OS) was calculated from the date of surgery to the date of death or the date of last follow-up. Disease-free survival (DFS) was calculated as the time from the date of surgery to the time of recurrence or date of last follow-up.

### Statistical analysis

Continuous variables were tested for normality by using the Kolmogorov-Smirnov test. Continuous data with a normal distribution were expressed as the mean ± standard deviation and compared using a standard *t* test. Otherwise, continuous data with non-normal distribution were compared using the Wilcoxon rank-sum test. Categorical variables were expressed as percentage and compared using the Chi-square test or Fisher's exact test as appropriate. Kaplan-Meier survival curves with log-rank tests and Cox proportional hazard regression analyses, recording patients at the time of last follow-up visit, were used to compare the OS and DFS rates. Variables with *P* < 0.1 in the univariate Cox regression analysis were progressed to a multivariate analysis using forward stepwise selection. All *P* values were two sided and a *P* value < 0.05 was considered to be statistically significant. Statistical analysis was performed using SPSS version 19.0 software (SPSS, Chicago, IL, USA) and MedCalc version 13.0.0.0 (MedCalc Software, Mariakerke, Belgium).

## SUPPLEMENTARY MATERIAL STROBE CHECKLIST


